# Venom-Induced Blood Disturbances by Palearctic Viperid Snakes, and Their Relative Neutralization by Antivenoms and Enzyme-Inhibitors

**DOI:** 10.3389/fimmu.2021.688802

**Published:** 2021-06-10

**Authors:** Abhinandan Chowdhury, Christina N. Zdenek, Matthew R. Lewin, Rebecca Carter, Tomaž Jagar, Erika Ostanek, Hannah Harjen, Matt Aldridge, Raul Soria, Grace Haw, Bryan G. Fry

**Affiliations:** ^1^ Venom Evolution Lab, School of Biological Science, University of Queensland, St. Lucia, QLD, Australia; ^2^ Department of Biochemistry & Microbiology, North South University, Dhaka, Bangladesh; ^3^ California Academy of Sciences, San Francisco, CA, United States; ^4^ Ophirex, Inc., Corte Madera, CA, United States; ^5^ Independent Researcher, Postojna, Slovenia; ^6^ Department of Companion Animal Clinical Sciences, Norwegian University of Life Sciences, Ås, Norway; ^7^ MicroPharm Limited, Newcastle Emlyn, United Kingdom; ^8^ Inosan Biopharma, Madrid, Spain

**Keywords:** venom, antivenom, enzyme inhibition, coagulopathy, snakebite

## Abstract

Palearctic vipers are medically significant snakes in the genera *Daboia, Macrovipera, Montivipera*, and *Vipera* which occur throughout Europe, Central Asia, Near and Middle East. While the ancestral condition is that of a small-bodied, lowland species, extensive diversification has occurred in body size, and niche specialization. Using 27 venom samples and a panel of *in vitro* coagulation assays, we evaluated the relative coagulotoxic potency of Palearctic viper venoms and compared their neutralization by three antivenoms (Insoserp Europe, VIPERFAV and ViperaTAb) and two metalloprotease inhibitors (prinomastat and DMPS). We show that variation in morphology parallels variation in the Factor X activating procoagulant toxicity, with the three convergent evolutions of larger body sizes (*Daboia* genus, *Macrovipera* genus, and *Vipera ammodytes* uniquely within the *Vipera* genus) were each accompanied by a significant increase in procoagulant potency. In contrast, the two convergent evolutions of high altitude specialization (the *Montivipera* genus and *Vipera latastei* uniquely within the *Vipera* genus) were each accompanied by a shift away from procoagulant action, with the *Montivipera* species being particularly potently anticoagulant. Inoserp Europe and VIPERFAV antivenoms were both effective against a broad range of *Vipera* species, with Inoserp able to neutralize additional species relative to VIPERFAV, reflective of its more complex antivenom immunization mixture. In contrast, ViperaTAb was extremely potent in neutralizing *V. berus* but, reflective of this being a monovalent antivenom, it was not effective against other *Vipera* species. The enzyme inhibitor prinomastat efficiently neutralized the metalloprotease-driven Factor X activation of the procoagulant venoms. In contrast, DMPS (2,3-dimercapto-1-propanesulfonic acid), which as been suggested as another potential treatment option in the absence of antivenom, DMPS failed against all venoms tested. Overall, our results highlight the evolutionary variations within Palearctic vipers and help to inform clinical management of viper envenomation.

## Introduction

Snakebite affects millions of people annually, killing over 100,000 and leaving many more with severe permanent injuries (1–3). Snake venom affects all physiological pathways reachable by the bloodstream, with blood coagulation itself a particular target (4). Despite their clinical importance, research into coagulotoxins (toxins that disrupt blood coagulation) has lagged behind other toxin types due to inherent difficulties of working with two enzyme systems (blood and venom) concurrently.

While snakebite in Europe is much less common than in African or Asiatic counties, it is still a potentially deadly medical emergency (5, 6). Common symptoms of envenomations from these vipers include local effects at the bite site such as swelling (edema), necrosis, and compartment syndrome (sometimes requiring fasciotomy), and/or systemic effects such as blood disturbances (coagulotoxicity), neuromuscular paralysis, myotoxicity, and hypotension (7–13). Severe envenomations can lead to amputation and/or death (7–13).

**Figure 1:**
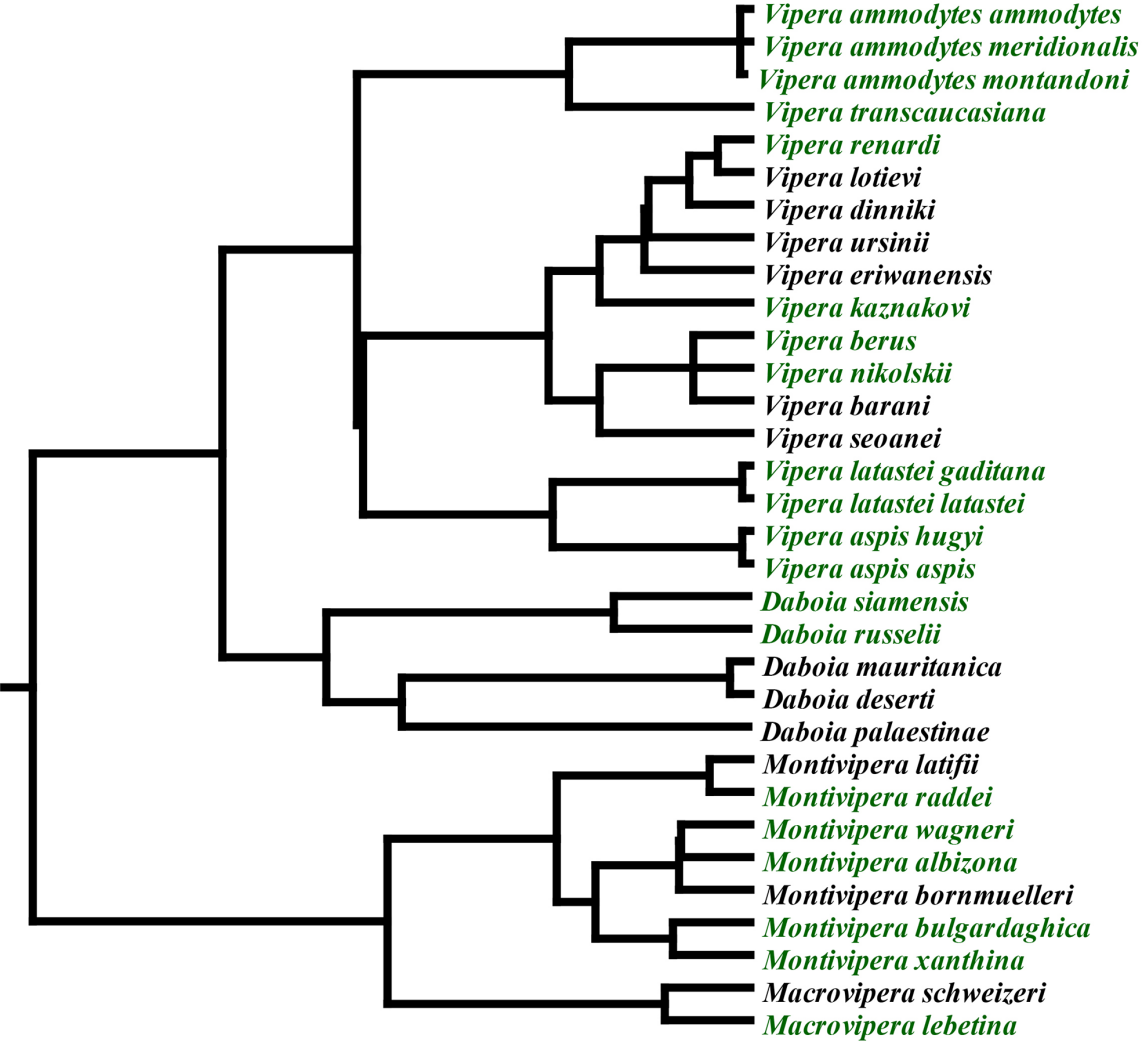
Phylogenetic relationships of Palearctic vipers (14). Green indicates species included in this study.

The Palearctic region is dominated by a clade of viperid snakes that emerged 20 million years ago, consisting of the genera *Daboia, Macrovipera, Montivipera*, and *Vipera* (**Figure 1** **),** with (*Daboia* + *Vipera*) being sister to (*Macrovipera* + *Montivipera*) (14). The basal morphology of this clade is small and species occupy lowland areas in arid regions. On two convergent occasions, giganticism evolved, once in *Daboia* and again independently in *Macrovipera*. Within *Vipera, V. ammodytes* is notably larger than other *Vipera* species.

*Daboia* and *Macrovipera* are well-characterised as causing potent procoagulant toxicity by converting the zymogen Factor X into the activated enzyme form FXa, which in turn converts prothrombin into thrombin, with the endogenous thrombin converting fibrinogen into fibrin, ultimately resulting in the development of well-ordered fibrin clots (15). The toxin class responsible for the coagulopathy produced by these snakes is a derived type of snake venom metalloprotease (P-IIId SVMP) that is characterised by two lectin toxins covalently linked to each other to form a dimer, with this dimer in turn covalently linked to the metalloprotease enzyme (16–18). In prey animals, this results in subjugation by thromboembolic stroke-induction and cardiovascular collapse from pulmonary embolism, but in human patients, the dilution of venom into a much larger blood volume results in formation of microemboli. While individual microemboli are of no clinical consequence, a net incoagulable state with morbidity and mortality results from the consumption of clotting factors in the process of venom-induced consumption coagulopathy (19).

**Figure 2:**
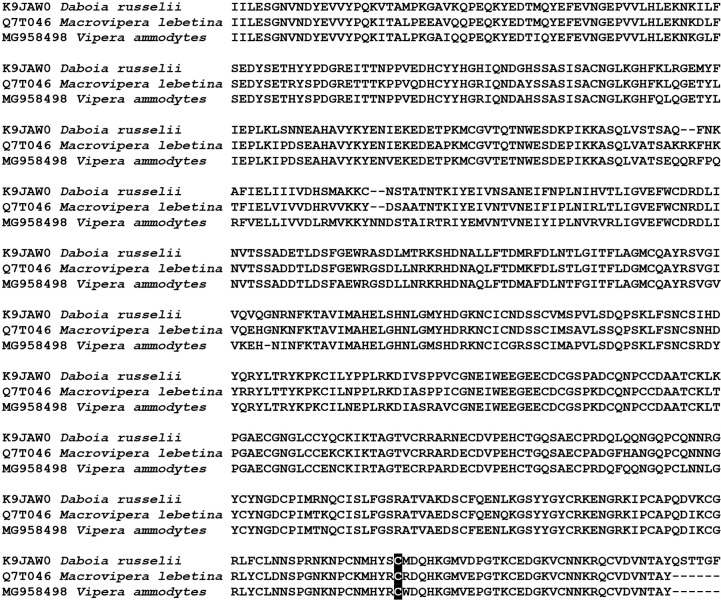
Sequence alignment of representative Factor X activating P-IIId SVMP characteristic of the Palearctic viper clade. Highlighted is the diagnostic cysteine that forms the interchain disulfide bond to a covalently linked lectin dimer. Uniprot (*Daboia* and *Macrovipera*) and Genbank (*Vipera*) accession codes are given for each sequence.

The genus *Vipera* is notable for being extremely widespread, radiating across Europe and Central Asia since its emergence 13 million years ago and having complex geographical histories (14, 20–22). Of particular clinical importance for human envenomings is coagulopathy (7–9, 12, 13). Documentation of Factor X activating P-IIId SVMP in at least one species (*V. ammodytes*) is consistent with its ability to produce severe coagulopathy (23). Sequence alignment shows that *Vipera* shares the characteristic cysteine used for the covalent linkage to the lectin dimer (**Figure 2**). The presence of FX activating P-IIId SVMP in *V. ammodytes* venom, like *Daboia* and *Macrovipera* venoms, combined with the observation of FX activation activity in *V. aspis* and *V. berus* venoms (24–26) and the documentation of congruent coagulopathy from *V. berus* envenomations (27) suggests that the presence of FX activating P-IIId SVMP is the basal state for the *Daboia*/ *Macrovipera*/ *Montivipera*/ *Vipera* clade.

The genus *Montivipera*, endemic to the Near and Middle East, is like *Vipera* in retaining the plesiomorphic small size but is notable for having uniquely specialized for montane habitats (28). The exception is *M. xanthina* which is derived relative to other species within this genus in being larger than the other species and radiating to occupy a lowland niche. Previous work has suggested that unlike the FX activating procoagulant basal state for the clade, this genus also has distinct anticoagulant venoms relative to the rest of the clade (29, 30) and induces systemic hemorrhage in victims rather than venom-induced consumptive coagulopathy (VICC) (31–34). The mechanism of action for this effect remains to be elucidated.

The divergence of venom documented for the *Daboia/Macrovipera/Montivipera/Vipera* clade indicates these snakes are an excellent model system to examine the influence of morphology and ecology on pathophysiological venom effects and how this influences clinical treatment options. Furthermore, the development of *in vitro* assays to replace *in vivo* assays to assess antivenom efficacy has been stated as one of the main issues to be addressed for the development and improvement of antivenoms (35). In this study, we tested a wide array of species (**Figure 1**), ascertained the relative effects upon blood clotting, whether this was mediated by Factor X activation, and the efficacy of three antivenoms and two repurposed enzyme inhibiting drugs. These results not only elucidated the evolutionary processes shaping venom diversification but also how this variation might influence the ability of therapeutics to neutralise pathophysiological effects.

## Materials and Methods

### Stocks Preparation

#### Venoms

All venom work was undertaken under the auspices of UQ IBSC approval *#*IBC134BSBS2015 and UQ NEWMA approval # 2021/AE000075. Pooled venoms (N = 3 captive adults unknown sex) which were immediately snap frozen in liquid nitrogen and kept at -80 until lypholisation and founder locality (if known) tested were: *Daboia russelii* (Pakistan), *D. siamensis* (Taiwan), *Macrovipera lebetina turanica* (Turkmenistan), *M. schweize*ri (Greece), *Montivipera albizona, M. bulgardaghica, M. raddei, M. wagneri, and M. xanthina (Turkey), Vipera ammodytes* (Krk Island, Croatia), *V. ammodytes* (Maribor, Slovenia), *V. ammodytes* (Ada Island, Montenegro), *V. ammodytes* (Lake Skadar, Montenegro), *V. ammodytes* (Slunj, Croatia), *V. a. meridionalis* (Greece), *V. a. montandoni* (Bulgaria), *V. aspis aspis* (France), *V. aspis hugyi* (Italy), *V. berus* (Norway), *V. berus* (Slovenia), *V. berus* (Snežnik Mountain, Slovenia), *V. kaznakovi* (Turkey), *V. latastei latastei* (Burgos, Spain), *V. latastei gaditana* (Spain), *V. nikolskii* (Russia), *V. renardi* (Russia), and *V. transcaucasiana* (Turkey). These lyophilized venoms were reconstituted to 1 mg/ml concentrated venom stock (concentration checked using 280 nm wavelength on a Thermo Fisher Scientific™ NanoDrop 2000 UV–Vis Spectrophotometer (Thermofisher, Sydney, Australia) following the manufacturer’s instructions 1 Abs = 1 mg/ml which is recommended when analyzing heterogenous solutions) by adding 50% glycerol and deionized water and stored at -20^o^C for further use.

#### Plasma

All plasma work was undertaken under the UQ IBSC approval #IBC134BSBS2015. Two bags of pooled 3.2 % citrated plasma (Label #A540020754341 & #A540020764777) were obtained from the Australian Red Cross (Research agreement #18-03QLD-09 and University of Queensland Human Ethics Committee Approval #2016000256). The two lots of plasma were pooled, aliquoted to 1 ml quantities, flash-frozen in liquid nitrogen, and stored at -80°C until required for testing. Aliquots were defrosted in at 37°C in a Thermo Haake ARCTIC water bath with a SC150-A40 circulator. Plasma aliquots were only used for up to an hour post-defrosting, after-which fresh aliquots were defrosted.

#### Fibrinogen

To ascertain the effect of venom on human fibrinogen clotting time, 100 mg of fibrinogen (Lot# SLBZ2294 Sigma Aldrich, St. Louis, Missouri, United States) was mixed with Owen Koller (OK) buffer (Stago catalogue #00360) to achieve a concentration of 4 mg/ml, then aliquoted to 1 ml quantities, flash-frozen, and stored at -80^o^C until further use. Defrosting steps and use were as per 2.1.2.

#### Antivenom (AV)

Antivenoms tested (and immunizing species for each antivenom) were as follows. Inoserp Europe (lot # 9IT03006), a 22.5 mg/ml F(ab′)_2_ antivenom made using an immunizing mixture consisting of *Macrovipera lebetina cernovi, M. l. obtusa, M. l. turanica, M. schweizeri, Montivipera xanthina, Vipera ammodytes, V. aspis, V. berus, and V. latastei*). MicroPharm VIPERFAV (lot #P4A281V), a 100 mg/ml F(ab′)_2_ antivenom made using an immunizing mixture consisting of *Vipera ammodytes, V. aspis, V. berus*). MicroPharm ViperaTAb (lot #VPT 002000), a 24.6 mg/ml Fab antivenom made using *V. berus* as the sole venom in the immunizing mixture. Both VIPERFAV and ViperaTAb came in a concentrate (1 X 4ml and 2 X 4ml vials respectively) solution, while Inoserp Europe was supplied in a lyophilized form, which was reconstituted with 10 ml of deionized water, according to company instructions. All antivenoms were centrifuged (RCF 14000) at 4°C for 10 min, followed by filtration of the supernatant (to remove insoluble material) using 0.45 μm Econofltr PES (Agilent Technologies, Beijing, China), aliquoted, and then stored at 4°C for future use. For tests (see 2.2.1.2), 5% AV solution was prepared by diluting with Owren Koller (OK) buffer (Stago catalogue #00360) for Inoserp, 4% for ViperaTAb, and 2% VIPERFAV. The percentages were calculated relative to the different antivenom treatment volume (10 ml, 8 ml, and 4 ml respectively) and thus the same proportion of each vial was used for the tests, thus allowing for a vial-to-vial comparison of efficacy. Therefore, calculating the ratios, the above-mentioned percentages were determined.

#### Enzyme Inhibitors

We set out to determine the efficacy of two small molecule inhibitors prinomastat hydrochloride ((S)-2,2-Dimethyl-4- ((p-(4-pyridyloxy) phenyl) sulfonyl) -3- thio- (catalogue# PZ0198) and DMPS 2,3-Dimercaptopropanesulfonic acid sodium salt monohydrate (catalogue # D8016) from Sigma-Aldrich. The powder was first dissolved in 10 % dimethyl sulfoxide (DMSO) and further diluted using deionized water to form 10 mM and 20mM stock solutions, respectively and stored at -80^o^C.

### Assay Conditions

#### Effects Upon Clotting Times of Plasma and Fibrinogen

##### Coagulotoxicity Effects on Plasma and Fibrinogen

Determination of venom effects upon coagulation was done using the STA-R Max® (Stago, Asnières sur Seine, France) coagulation analyser and adapted from validated coagulation assay protocols (36–39). 1mg/ml venom stocks (50% glycerol/50% deionized water) were diluted to 100 μg/ml with OK Buffer (Stago catalogue #00360) to prepare the working stock, which was later loaded into the analyser for running 8-point concentration curves with serial dilutions of 1, 1/2, 1/5, 1/12.5, 1/30, 1/80, 1/160, and 1/400 (final reaction concentrations of venom). In an automated process, 50 µl venom stock (100 µg/ml starting concentration and serially diluted to form final reaction concentrations as noted above) were added to a cuvette, followed by the addition of 25 µl of OK buffer, 50 µL of 0.025 M calcium chloride (Stago catalogue # 00367), and 50 µl of phospholipid (Stago catalogue #00597), and then the mixture incubated for 2 minutes at 37°C. Subsequently 75μl of plasma or 75μl of 4 mg/ml fibrinogen was added after incubation, and clotting time was recorded immediately. To avoid abnormal results due to venom degradation, venom was changed after each set. As a positive control, coagulation activator kaolin (Stago C·K Prest standard kit, Stago catalogue #00597) was used to check for consistent plasma responses, and 25 µL of the thrombin (Stago catalogue #115081) as a positive control to check for consistent plasma responses. The negative control for both plasma and fibrinogen studies was 50% glycerol/deionized water that was diluted to the same amount as the venom 50% glycerol venom and positive control stocks (1% final concentration for venom, positive controls, and negative controls).

##### Vial to Vial Antivenom Efficacy and Enzyme-Inhibitor Efficacy

Efficacy of antivenoms or enzyme inhibitors in neutralizing toxic effects of venom upon blood clotting was tested by repeating the above mentioned 8-point concentration curves, but the 25 µl of OK buffer (added to the cuvette before incubation) was replaced with 25 µl of antivenom (final reaction concentration of Inoserp Europe 0.5%, ViperaTAb 0.4%, and VIPERFAV 0.2%. The differences in concentration were reflective of the different antivenom vial size (10 ml, 8 ml, and 4 ml respectively) and thus the same proportion of each vial was used for the tests, thus allowing for a vial-to-vial comparison of efficacy. Similarly, enzyme-inhibitors at 2 mM working stock for prinomastat and DMPS, (final reaction concentration of 0.2 mM) was tested against *Daboia russelii* (Pakistan), *D. siamensis* (Taiwan), *Macrovipera lebetina turanica* (Turkmenistan), *M. schweize*ri (Greece), *V. ammodytes* (Ada Island, Montenegro), *V. aspis hugyi* (Italy).

##### Thromboelastography

To evaluate the strength of the clot and total thrombus generated by the venoms in plasma, further investigation was carried out by using TEG5000 haemostasis analyzers (Haemonetics®, Haemonetics.com, catalogue # 07- 033). The assay included consecutive addition of 72 μl of 0.025M CaCl_2_, 72 μl phospholipid, 20 μl of the OK buffer, 7 μl of 1 mg/ml of venom, and 189 μl plasma to the reaction cup, followed by automated measurement. For spontaneous clotting of plasma (negative control), 7 μl 50% deionized water/glycerol was replaced with venom. Similarly, 7 μl of thrombin (Stago catalogue #115081 Liquid Fib) or 7 μl Factor Xa (Stago catalogue #253047 Liquid Anti-Xa) was replaced with venom, for two positive controls. Each reaction ran for 30 minutes.

##### Clotting Factor Activation Assays

Fluoroskan Ascent™ (Thermo Scientific, Vantaa, Finland) was employed to detect Factor X and prothrombin activation based on the results of the above methods (38, 39). **Table 1** reagents were manually plated in 384-well plates (black, lot#1171125, Nunc™ Thermo Scientific, Rochester, NY, USA), followed by automated pipetting of 70 μl of buffer containing 5 mM CaCl2,150 mM NaCl, and 50 mM Tris-HCl (pH 7.3) and Fluorogenic Peptide Substrate, (ES011Boc-Val-Pro-Arg-AMC. Boc: t-Butyloxycarbonyl; 7-Amino-4-methylcoumarin; R & D systems, Cat# ES011, Minneapolis, Minnesota) in 500:1 ratio to start the reaction; with the plate warmed up at 37°C and shaken for 3 s before each measurement. The reaction was carried out 300 times at 390/460 nm (excitation/emission) and every 10 s the fluorescence generated by the cleavage of the substrate was measured by Ascent® Software v2.6 (Thermo Scientific, Vantaa, Finland). To obtain results, blank (background) values were subtracted from all other reactions, followed by subtraction of “venom without zymogen” values from “venom with zymogen” values (to nullify artificial increment of the fluorescence values caused some venoms which work directly on the substrate). Finally, the resultant values from the subtractions were normalized as a percentage relative to FXa or thrombin by organizing in Excel and then analysing in GraphPad PRISM 8.1.1 (GraphPad Prism Inc., La Jolla, CA, USA).

**Table 1:** Fluorescent substrate activation assay.

Blank	20 μl of enzyme buffer without calcium (150 mM NaCl, and 50 mM Tris-HCl (pH 7.3) + 10ul PPL)
Control with Activated Enzyme	10μl of enzyme buffer without calcium (150 mM NaCl, and 50 mM Tris-HCl (pH 7.3) + 10ul PPL + 10μl (10 μg/ml FXa (Haematologic Technologies catalog # GG0621) or 1 μg/ml Thrombin (Haematologic Technologies catalog # JJ0701))
Control with Zymogen	10μl of enzyme buffer without calcium (150 mM NaCl, and 50 mM Tris-HCl (pH 7.3) + 10ul PPL + 10μl (10 μg/ml FX (Haematologic Technologies catalog # HH0821) or 1 μg/ml prothrombin (Haematologic Technologies catalog # HH1010))
Venom without Zymogen	10μl of enzyme buffer without calcium (150 mM NaCl, and 50 mM Tris-HCl (pH 7.3) + 10ul PPL + 10μl venom
Venom with Zymogen	10μl zymogen (10 μg/ml FX or 1 μg/ml prothrombin) + 10ul PPL + 10μl venom (1 μg/ml FX (Haematologic Technologies catalog # HH0821) or 0.1 μg/ml prothrombin (Haematologic Technologies catalog # HH1010))

##### Clotting Factor Inhibition Assays

Samples were diluted at 1:10 with OK buffer. 50 μl of venom + 50 μl of CaCl_2_ (0.025M, Stago Cat#11851) + 50 μl of phospholipid solution (STA C.K. Prest standard kit, Stago Cat#12207, solubilized in 5 ml OK buffer) + 25 μl of clotting factor (thrombin, FIXa, FXa, FXIa, and FXIIa) were incubated for 120s at 37°C. Subsequently 75 μl of plasma was added, and clotting time was measured. OK buffer control was used as a negative control.

### Statistical Analyses

All tests were run in triplicate. All data plotting and statistical analysis were done by using GraphPad PRISM 8.1.1 (GraphPad Prism Inc., La Jolla, CA, USA). Determination of the AV efficacy against venom, the area under the curve (AUC) for both venom and venom + antivenom was calculated using the software, followed by generation of X-fold magnitude of shift in Excel (formulae [(AUC of venom incubated with antivenom/AUC of venom) - 1]). The resulting values for X-fold magnitude of shift; if 0, indicated no neutralization (no change of clotting time curve), and if over 0, demonstrated venom neutralization (change in clotting time curve).

## Results

### Effects Upon Clotting Times of Plasma and Fibrinogen

#### Coagulotoxicity Effects

**Figure 3:**
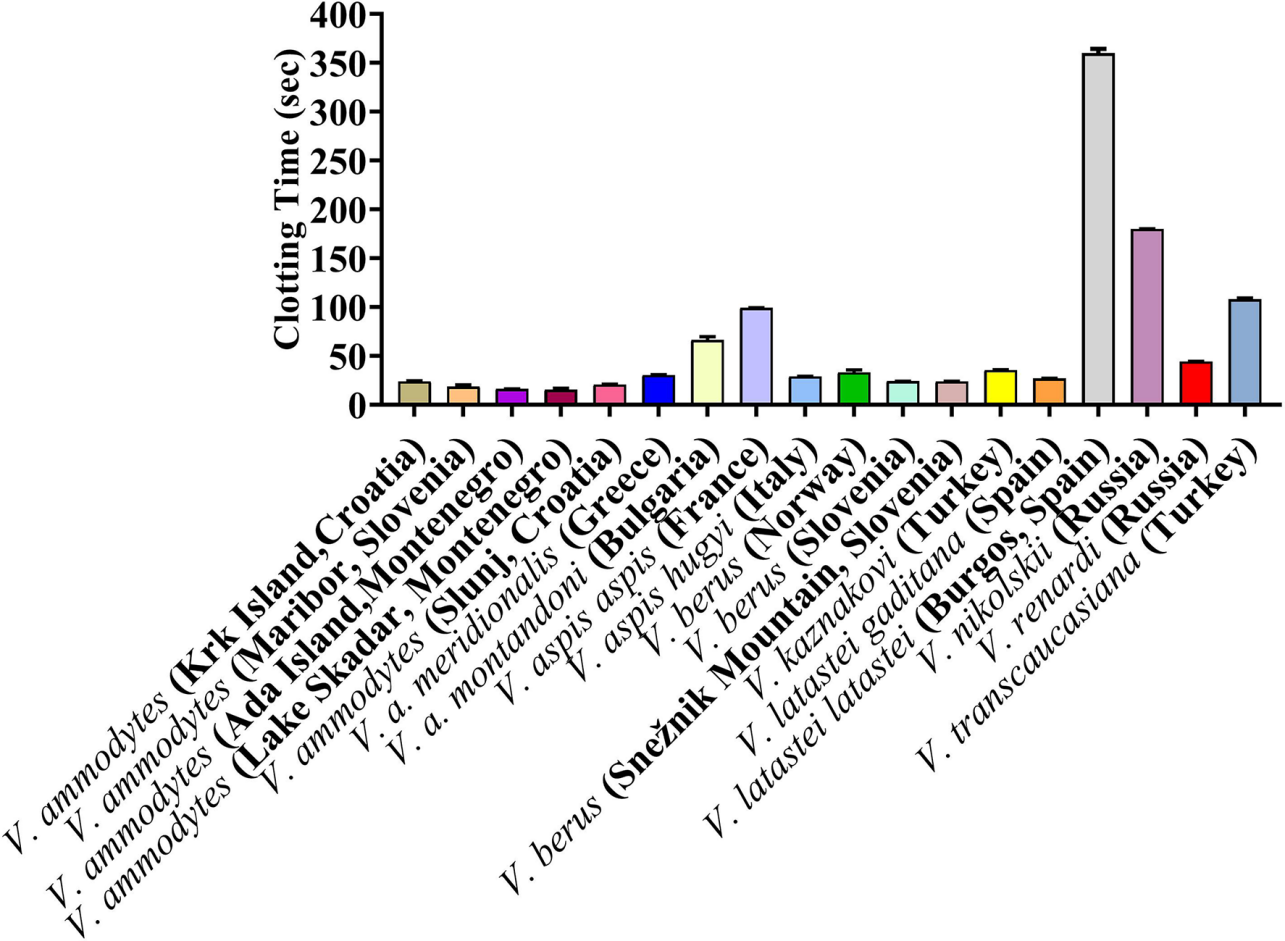
Clotting times on human plasma produced by *Vipera* venoms (20 µg/ml).

The *Vipera* venoms all displayed a clotting effect upon plasma, but with a wide range of values. The maximum velocity clotting times (in seconds) at the highest concentration (20 μg/ml) of (seconds +/- SD) (with smaller numbers indicating stronger effect) was: 15.4 +/- 0.9 *V. ammodytes* (Lake Skadar, Montenegro); 16.0 +/- 0.1 *V. ammodytes* (Ada Island, Montenegro); 18.4 +/- 1.9 *V. ammodytes* (Maribor, Slovenia); 20.4 +/- 0.5 *V. ammodytes* (Slunj, Croatia); 23.0*+/-* 0.6 *V. ammodytes* (Krk Island, Croatia); 23.9 +/- 0.1 *V. berus* (Slovenia); 23.9 +/- 0.1 *V. berus* (Snežnik Mountain, Slovenia); 26.7 +/- 0.3 *V. latastei gaditana* (Spain); 28.7 +/- 0.2 *V. aspis hugyi* (Italy); 30.3 +/- 0.4 *V. a. meridionalis* (Greece); 33.0 +/- 2.4 *V. berus* (Norway); 35.3 +/- 0.4 *V. kaznakovi* (Turkey); 44.1 +/- 0.4 *V. renardi* (Russia);66.2 +/- 3.4 *V. a. montandoni* (Bulgaria); 98.9 +/- 0.3 *V. aspis aspis* (France);107.8+/- 1.4 *V. transcaucasiana* (Turkey); 179.7 +/- 0.3 *V. nikolskii* (Russia); and 359.8 +/- 4.5 *V. latastei latastei* (Spain) (**Figure 3**). The plasma kaolin positive control was 51.0 +/- 0.34, while the negative control (spontaneous clotting) was 645.2 +/- 9.8

To check significant differences the values One Way ANOVA was carried out on the clotting times. There were no significant differences (*p* > 0.05 at 95.00 % confidence interval) between *V. ammodytes* (Krk Island, Croatia) versus (*vs.*), *V. ammodytes* (Slunj), Croatia) *V. berus* (Slovenia), *V. berus* (Snežnik Mountain, Slovenia) and *V. latastei gaditana* (Spain); *V. ammodytes* (Maribor, Slovenia) *vs. V. ammodytes* (Ada Island, Montenegro), *V. ammodytes* (Lake Skadar, Montenegro) and *V. ammodytes* (Slunj, Croatia); *V. ammodytes* (Ada Island, Montenegro) *vs. V. ammodytes* (Lake Skadar, Montenegro) and *V. ammodytes* (Slunj, Croatia); *V. ammodytes* (Slunj, Croatia) *vs. V. berus* (Slovenia), *V. berus* (Snežnik Mountain, Slovenia); *V. a. meridionalis* (Greece) *vs. V. aspis hugyi* (Italy), *V. berus* (Norway) and *V. latastei gaditana* (Spain); *V. aspis hugyi* (Italy) *vs. V. berus* (Slovenia), *V. berus* (Norway) and *V. latastei gaditana* (Spain); *V. berus* (Norway) *vs. V. kaznakovi* (Turkey); *V. berus* (Slovenia) *vs. V. berus* (Snežnik Mountain, Slovenia) and *V. latastei gaditana* (Spain); *V. berus* (Snežnik Mountain, Slovenia) *vs. V. latastei gaditana* (Spain), while there were significant difference between clotting times between all other vipers.

However, like shown previously for *Daboia* and *Macrovipera* venoms (38) none of the *Vipera* venoms clotted fibrinogen with the assay measurements reaching the machine maximum of 999 seconds. The clotting time for the thrombin positive control was (seconds +/- SD) 3.6 +/- 0.1 seconds. This suggested that the clotting action shown for plasma was due to the activation of a clotting factor, which was explored further (see 3.3 below).

In contrast, all *Montivipera* venoms demonstrated potent anticoagulant actions on plasma, with the test sets all reaching the machine maximum reading time of 999 seconds. This included the derived *M. xanthina* which has secondarily colonized a lowland habitat relative to the other species in this clade (40) and also evolved a larger body size. Despite these derivations, it retains the potent anticoagulant venom characteristic of the *Montivipera* genus.

#### Antivenom and Enzyme Inhibitor Efficacy

**Figure 4:**
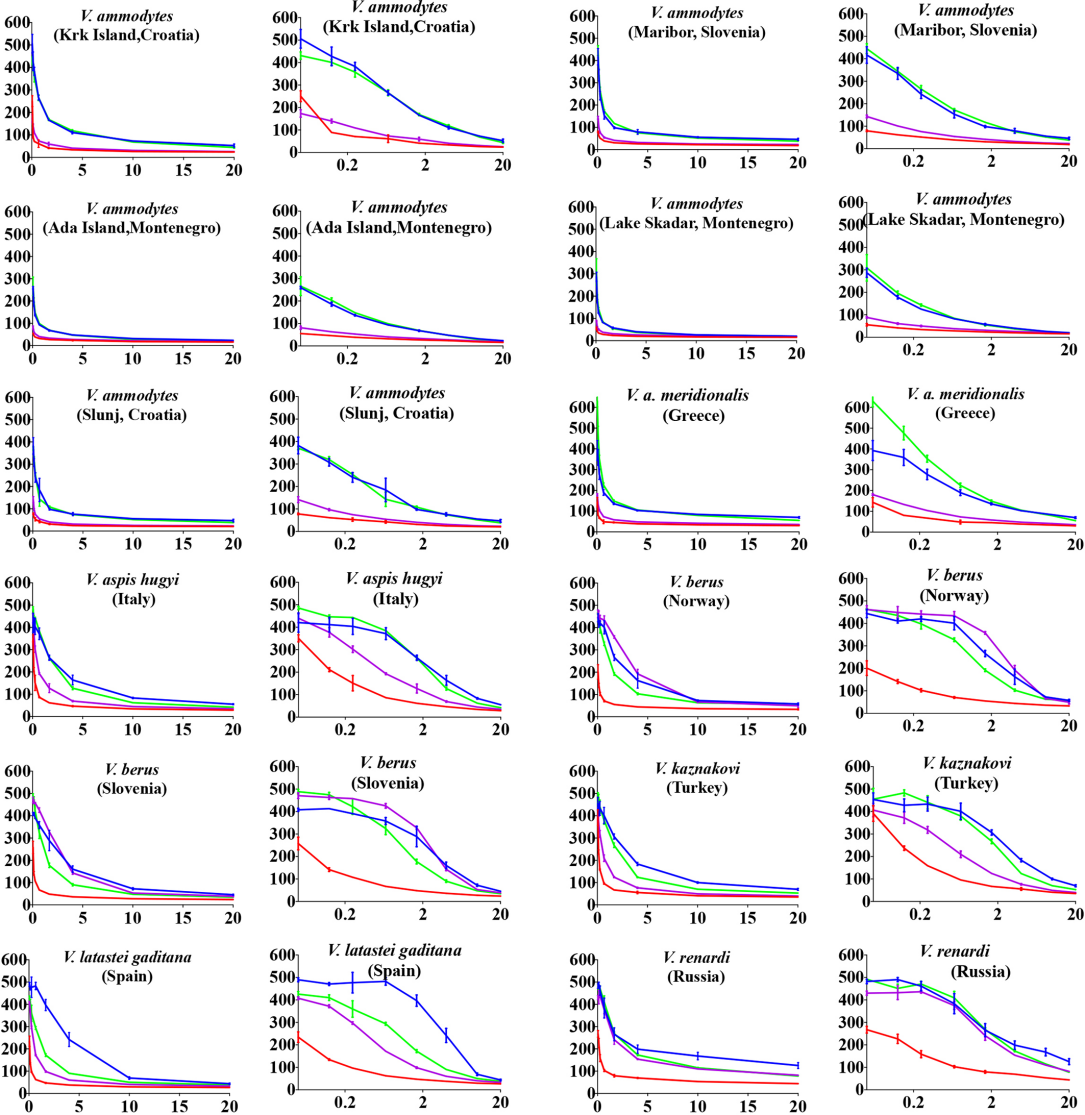
8-point concentration curves, x-axis showing concentrations of venom in μg/ml and y-axis showing clotting times in seconds of human plasma with venom and relative antivenom efficacy. For each species, linear graphs are presented on the left and logarithmic views on the right. Shown are: venom-induced clotting times (red curves); venom-induced clotting times after preincubation with Inoserp AV (final concentration 0.5%; spontaneous control- 420.2 +/27.7) (blue curves), ViperaTAb AV (final concentration 0.4%; spontaneous control- 478.4 +/33.3) (purple curves), or VIPERFAV AV (final concentration 0.2%; spontaneous control- 509.7 +/13.5) (green curves). Values are mean ± SD of N = 3 and shown as dots with error bars. Some error bars are too small to see.

**Figure 5:**
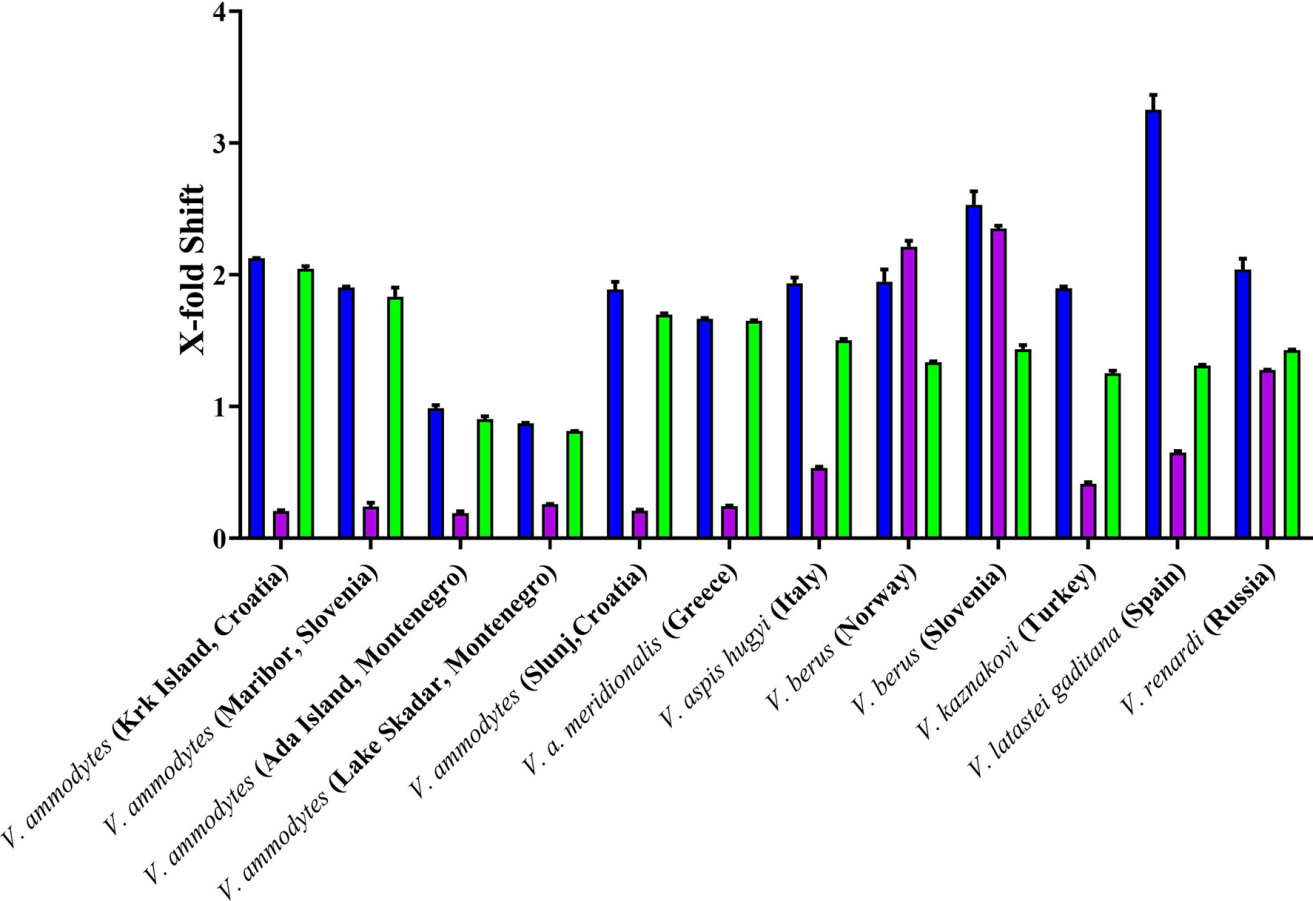
X-fold magnitude of shift of plasma clotting time due to incubation of antivenoms indicated by: Inoserp in blue bars, ViperaTAb in purple bars; and VIPERFAV in green bars. X-fold magnitude of shift was calculated by the formula [(AUC of antivenom + venom/AUC of venom) -1]. A value of 0 indicates no shift (no neutralization by antivenom), while a value above 0 indicates neutralization by antivenom. Values are mean ± SD of N = 3.

Inoserp and VIPERFAV were comparable against all *V. ammodytes* populations and *V. a. meridionalis*, while VIPERFAV was moderately less effective for other species, except for *V. latastei gaditana* against which it performed comparatively poorly (**Figures 4** and **5**). Interestingly, both Inoserp and VIPERFAV, which had *V. ammodytes* as their immunizing species, showed lower level potency against *V. ammodytes* species hailing from Montenegro compared to other *V. ammodytes* populations. Consistent with ViperaTAb having only *V. berus* as an immunizing species, it performed extremely well against *V. berus* but compared to the other two antivenoms, it performed poorly against the other species except for moderate levels of neutralization of *V. renardi*.

**Figure 6:**
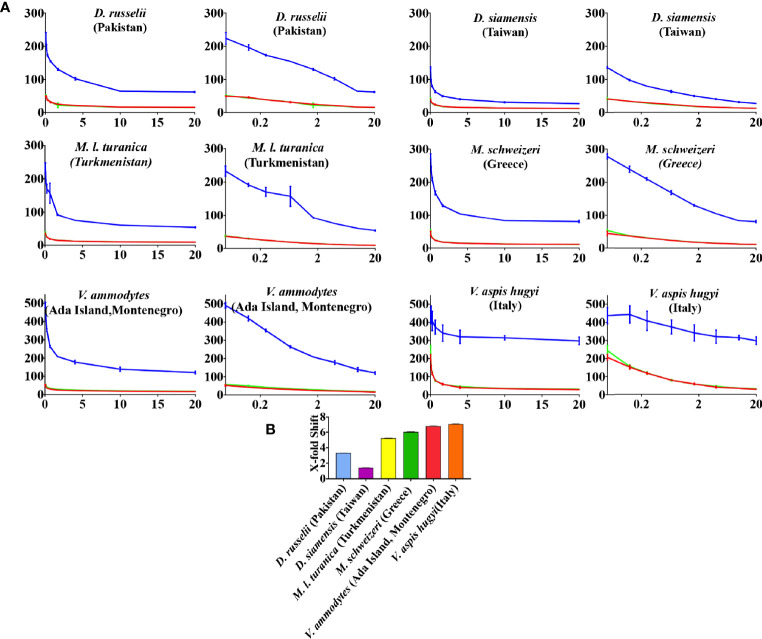
**(A)** 8-point concentration curves, x-axis showing concentrations of venom in μg/ml and y-axis showing clotting times in seconds of human plasma with venom and relative inhibitor efficacy. For each species, linear graphs are presented on the left and logarithmic views on the right. Shown are venom-induced clotting times (red curves), effect of venoms after preincubation with prinomastat (final concentration 0.2 mM%; spontaneous control- 484.8 +/- 11.0) (blue curves), and effect of venoms after preincubation with DMPS (final concentration 0.2 mM %; spontaneous control- 425.8 +/3.3) (green curves). Values are mean ± SD of N = 3 and shown as dots with error bars. Some error bars are too small to see and the failure of DMPS to shift the curves results in an identical line to the red (venom only) curves. **(B)** Bar graphs of X-fold magnitude of shift of plasma clotting time due to induction of prinomastat. X-fold magnitude of shift was calculated by the formula [(AUC of inhibitor + venom/AUC of venom) -1]. A value of 0 is no shift (no neutralization by inhibitor), while a value above 0 indicates neutralization by inhibitor. Values are mean ± SD of N = 3.

While the antivenoms had variable differences, prinomastat highly neutralized not only *Vipera* representatives but also *Daboia* and *Macrovipera* (major metalloprotease dependent venoms) representatives at 0.2 mM concentration (**Figure 6**). In contrast, DMPS performed extremely poorly against all venoms (**Figure 6**).

### Thromboelastography

**Figure 7:**
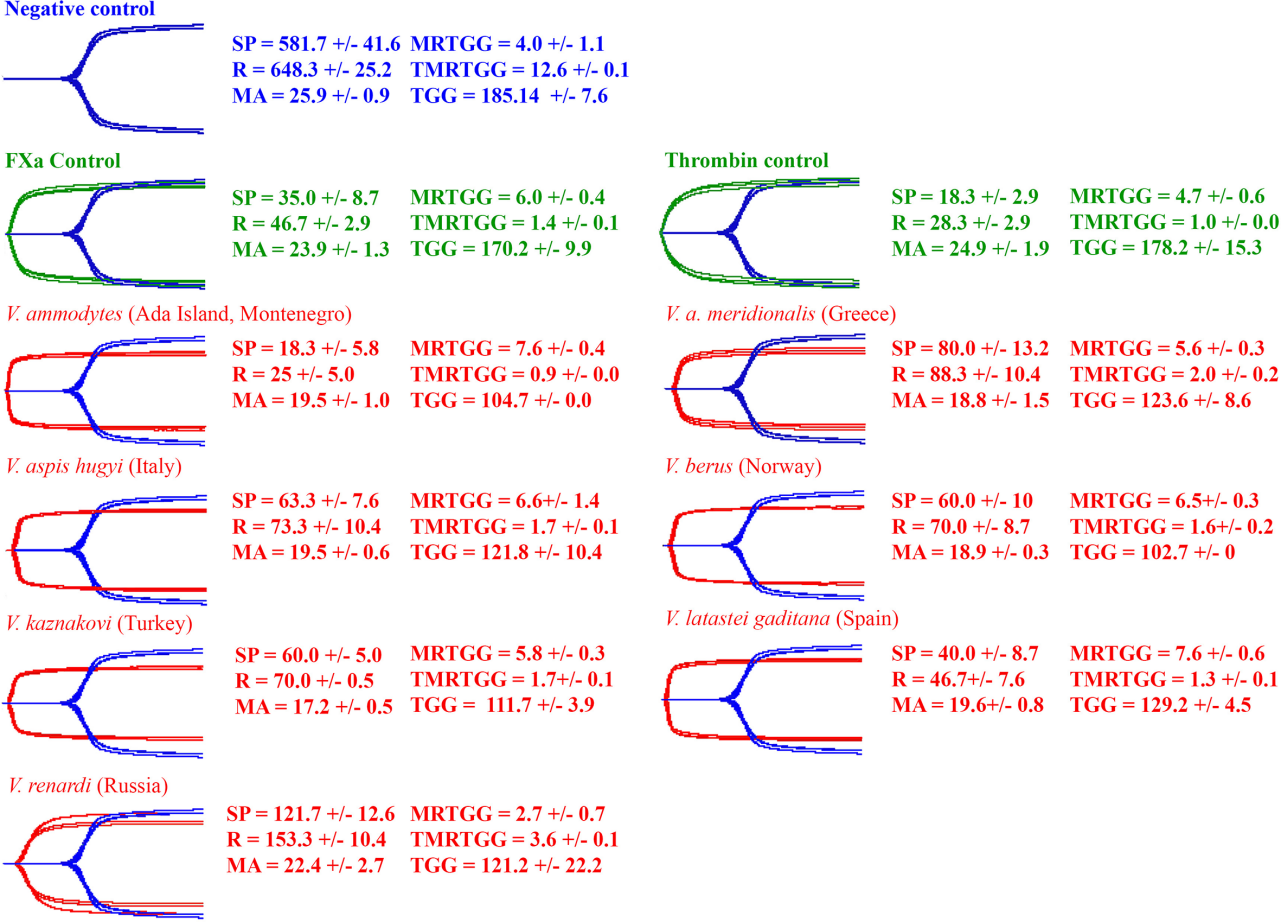
Overlaid thromboelastography traces of spontaneous clotting negative control (blue), Factor X and thrombin positive controls (green) and venom-induced clotting experimental condition (red). Parameters: SP = the split point (time till clot formation begins) (sec); R = time until detectable clot (2 mm +) is formed (sec); MA = maximum amplitude of clot (mm); MRTG = maximum rate of thrombus generation (dynes/cm2/s); TMRTG = time to maximum rate of thrombus generation (min); and TGG = total thrombus generated (dynes/cm2). Values are mean ± SD of N = 3.

Consistent with the activation of a clotting factor and the resulting generation of endogenous thrombin, all venoms produced strong, stable clots in the thromboelastography assays (**Figure 7**).

### Clotting Factor Zymogen Activation

**Figure 8:**
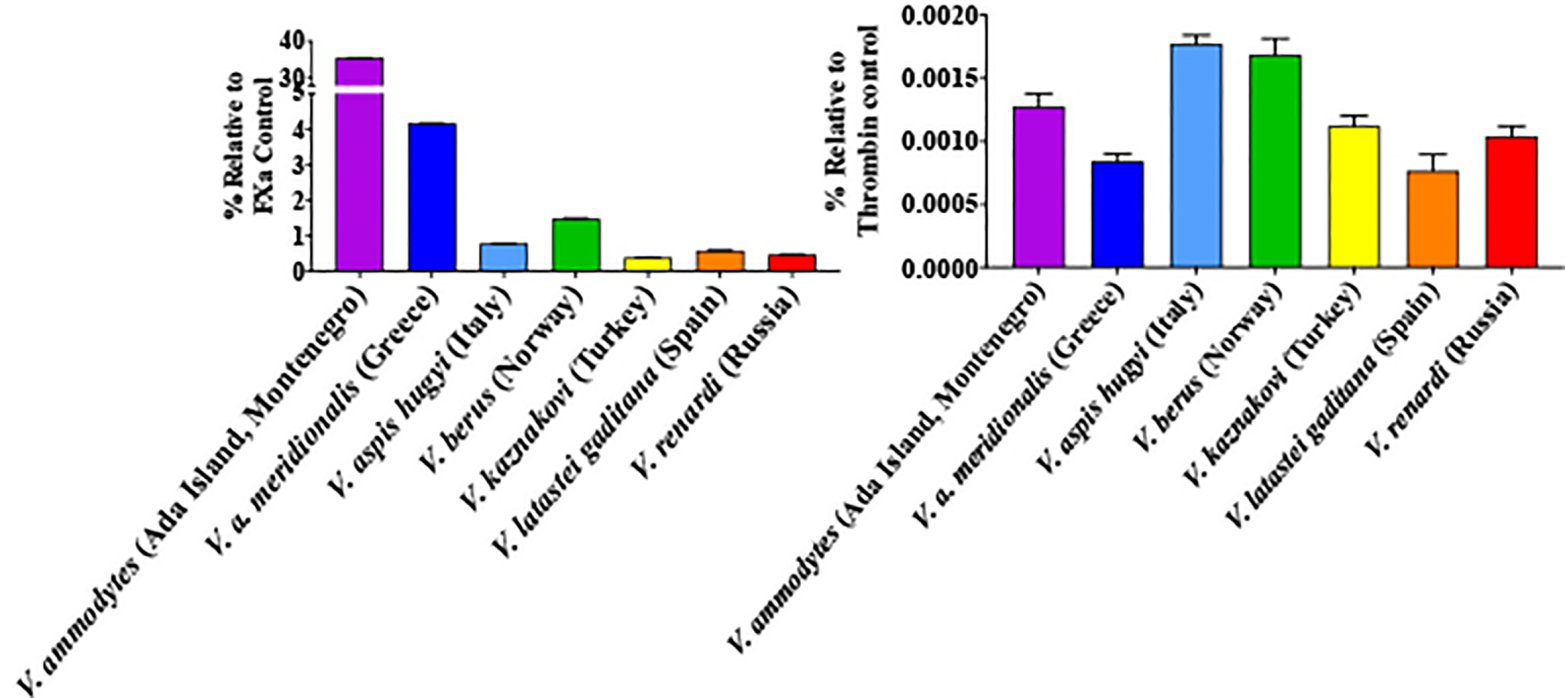
Ability of venoms to activate Factor X (left) compared to prothrombin (right). Results are presented relative to the control of the same amount of the corresponding activated enzyme form (note difference in y-axis scales between the two graph sets). Data points are N = 3 mean ± SD.

All venoms displayed the ability to activate Factor X but only negligible activation of prothrombin (**Figure 8**). The relative potencies were congruent with the action of respective venoms on plasma (**Figures 3** and **7**). *V. ammodytes* (Ada Island, Montenegro), which was the fastest on plasma, showed the highest activation of FX. Conversely, *V. renardi* was the slowest on plasma and also activated FX the least.

### Clotting Factor Inhibition

**Figure 9:**
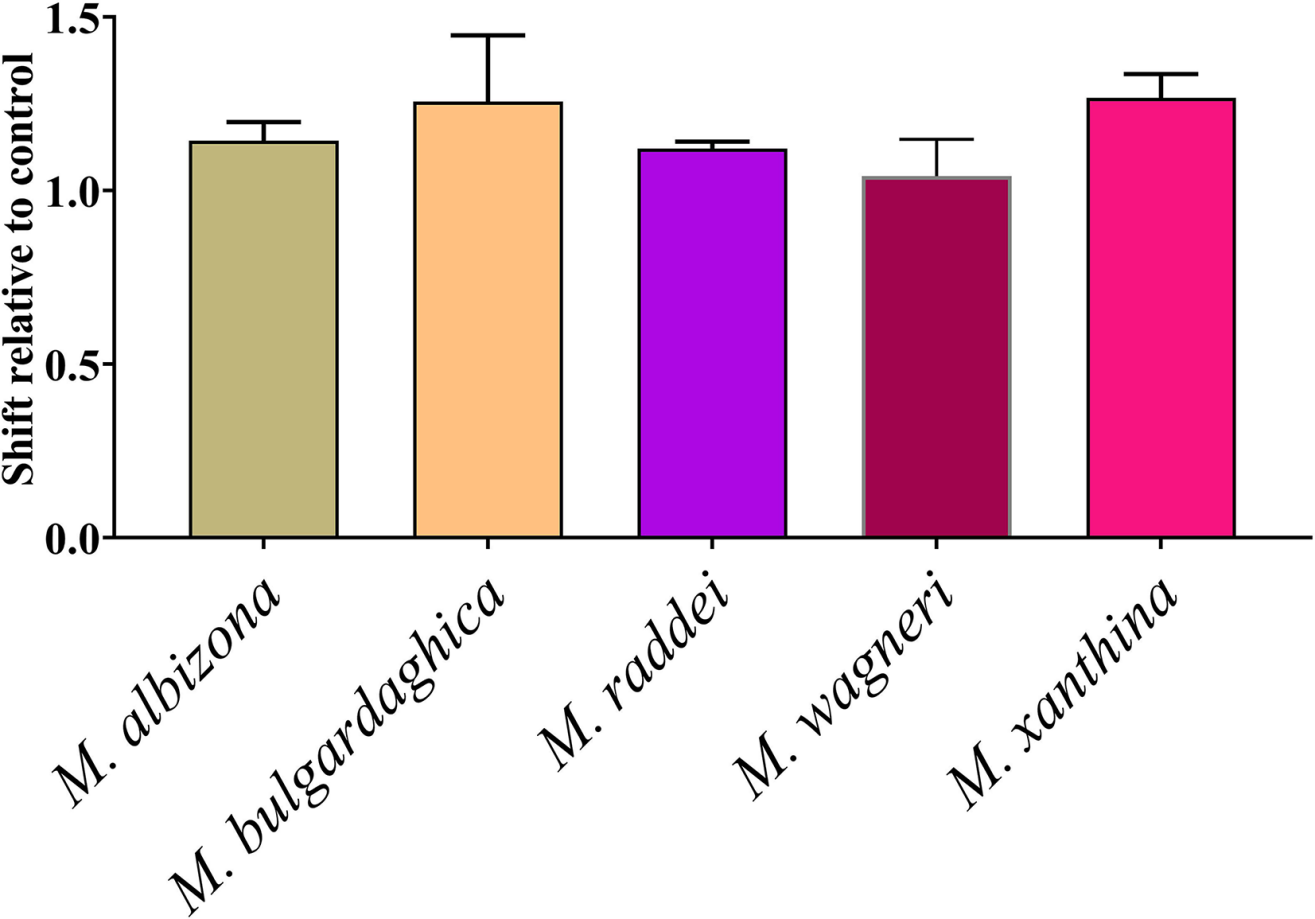
Shift of clotting time relative to control due to inhibition of Factor Xa (no effect relative to control would be a value of 0). Data points are N = 3 mean ± SD.

As the *Montivipera* venoms were shown to be potently anticoagulant, tests were undertaken to ascertain if the inhibition was due to the inhibition of thrombin, FIXa, FXa, FXIa, or FXIIa. Only FXa was shown to be inhibited, with all the species having this action at comparable potency (**Figure 9**).

## Discussion

Our study set out to examine changes in venom biochemistry relative to two distinct types of derivation in this clade away from the diminutive, low-land niche occupying last common ancestor: that of the three convergent evolutions of giganticism (*Daboia* genus, *Macrovipera* genus, and *Vipera ammodytes* as the significantly largest member of the *Vipera* genus); and the occupation of the high-land niche by the *Montivipera* genus and *Vipera latastei latastei* as a unique high-land specialist within the *Vipera* genus.

Consistent with previous results demonstrating that *Daboia* and *Macrovipera* species have extremely potent procoagulant (Factor X activating) venoms (38), this study revealed that the largest *Vipera* species (*V. ammodytes*) was also the most potent *Vipera* in activating Factor X, with one population (Lake Skadar, Montenegro) even approaching that of *Daboia* and *Montivipera* speed of action. Consistent with the link between size and relative FX activation levels of the venoms, the more diminutive sister species *V. transcaucasiana* and all other smaller *Vipera* species were comparably less potent than *V. ammodytes.* This is in contrast to other snakes, such as the *Bitis* genus in which gigantism evolved on two separate occasions (41), neither of which were linked to notable changes in coagulotoxicity (42).

The strongest divergence in venom action was for the *Montivipera* species. The diversification into a unique high-altitude niche relative to the low-altitude last common ancestor of the Palearctic viper clade was accompanied by a change from the procoagulant ancestral trait to potent anticoagulant toxicity. Specifically, instead of activating Factor X into Factor Xa like the last common ancestor of the Palearctic viper clade, the venom of these snakes was shown in this study to inhibit Factor Xa. This radical change in venom biochemistry was retained in *M. xanthina.* This is significant as this species is nested deep within the *Montivipera* genus (**Figure 1**), and has secondarily evolved to occupy a low-land niche from within this montane specialist genus (40). However, the relatively recent shift from high-land to low-land niche has not yet been accompanied by a change in venom biochemistry. The relationship between a secondary losses of the ancestral procoagulant trait accompanying the specialization for a high-land niche was reinforced by the convergent action within *V. latastei*, which has two subspecies: the low-land *V. latastei gaditana* and the high-altitude (2,900 to 3,600 feet) subspecies *V. latastei latastei. V. l. gaditana* is like other *Vipera* in having a FX activating procoagulant venom, while *V. latastei latastei* has lost the procoagulant trait (21).

Antivenom neutralization patterns broadly followed the immunizing mixtures. Reflective of having *V. berus* in the immunizing mixture, ViperaTAb was strongly effective only against the *V. berus* venoms. The multi-species immunizing mixture of VIPERFAV (*Vipera ammodytes, V. aspis, V. berus*) was reflected in its multi-species neutralisation potential. Consistent with Inoserp Europe having a very complex immunizing mixture (the *Vipera* species *V. ammodytes, V. aspis, V. berus*, and *V. latastei*, in addition to the *Macrovipera* species *M. lebetina cernovi, M. schweizeri, M. l. obtusa, M. l. turanica* and the *Montivipera* species *M. xanthina*), it displayed the greatest paraspecificity. Consistent with the variation between species and populations within a species observed in this study, previous work using other methods to ascertain variations in other characteristics have similarly reported such variations (43–47). The discrepancy of antivenom neutralizing property on the venom of same species from different locations or within genus is evident in other studies, which is a major issue in antivenom production (42, 48–58).

The comparative testing of the enzyme inhibitors prinomastat and DMPS revealed highly contrasting differences in their specific abilities to neutralize the P-IIId SVMP responsible for FX activation by the venoms in this study. This is congruent with recent data published for FX activation of neonate *Crotalus culminatus* venom, which prinomastat neutralised but DMPS at the same concentration did not (59) and another report of DMPS requiring long incubation times to exert a discernable effect (60).

Additional important caveats are that this study only examined the neutralization capacity of antivenoms and enzyme-inhibitors using *in vitro* systems. Neutralization assays conducted in this study were under preincubation conditions and as such, demonstrated failure under such idealized conditions are strongly suggestive of failure under dynamic physiological conditions. Thus the limited taxonomical range of ViperaTAb antivenom in this study is strongly suggestive of limited taxonomical range when tested *in vivo* and real-world use, as is the failure of DMPS suggestive of *in vivo* and real-world failure. However, success under such *in vitro* conditions does not automatically translate into success in *in vivo* models or real-world clinical cases. Therefore additional studies must be undertaken using *in vivo* assays and then clinical studies before clinical recommendations can be made. Thus while the Inoserp and VIPERFAV antivenoms, and the enzyme-inhibitor prinomastat, are predicted by this study to have broad utility, this must be corroborated by *in vivo* and subsequent clinical studies.

Another important caveat is that this study focused solely upon the coagulotoxic effects of these venoms. Each of these snake venoms has clinically relevant profile extending beyond coagulopathy, such as neurotoxicity, another major lethal trait (61). In addition to P-IIId SVMP, these venoms contain myriad other toxins, including snake venom serine protease (SVSP), phospholipase A_2_ (PLA_2_), and L-Amino acid oxidase (LAAO) (62). In a study on *V. berus* venom, the complex presence of basic phospholipases, SVSPs, LAAO, SVMP, were responsible for hemotoxicity, myotoxicity, cytotoxicity and neurotoxicity (63). *V. aspis* shared similar toxins with *V. berus* along with PLA_2_ (ammodytoxin B-like PLA_2_: neurotoxic effect), SVMP inhibitor, SVMP, SVSP, and disintegrins; however, a higher presence of disintegrins were seen in *V. aspis* compared to *V. berus* (64, 65). In separate studies on *V. kaznakovi* and *V. anatolica* venom, an abundance of SVMP was evident. However, *V. kaznakovi* had a higher percentage of PLA_2_ and SVSP compared to *V. anatolica* (46, 66). *V. nikolskii*, *V. orlovi*, *V. renardii*, and *V. kaznakovi* were all reported to have greater PLA_2_ percentage compared to other toxins followed by SVMPs (67–69). Great diversity may exist within each of these toxin classes. For example, a SVSP isolated from *V. ammodytes* venom (VaaSP-VX) has been shown to activate Factor X (FX) and Factor V (FV) simultaneously, a function congruent with the metalloproteases in this study (although this toxin is in much lower levels in the venom than the metalloprotease), while another *V. ammodytes* SVSP (VaF1 toxin) has α-fibrinogenolytic activity (70, 71). Another *V. ammodytes* toxin, a myotoxic secreted PLA_2_ analogue ammodytin L (AtnL) was reported to cause irreversible atrioventricular (AV) blockade (72, 73). *V. ammodytes meridionalis* has been shown to share similar toxins as well as vipoxin (PLA_2_, postsynaptic neurotoxin) (74). The presence of additional toxin actions beyond those examined in this study is reflected in complex envenomation clinical profiles (61). Thus, more comprehensive *in vitro* assays and also *in vivo* studies must be conducted before clinical recommendations can be finalised regarding treatment options for particular species or populations within a species.

This work provides data useful for predicting potential clinical effects and contributing to the evidence-based design of clinical management strategies. As with *Daboia* and *Macrovipera*, an increase in *Vipera* species sizes was correlated with an increase in the FX activation potency, with the largest species (*V. ammodytes*) possessing the most potently procoagulant venom. Consistent with their multi-species immunizing mixture, both Inoserp Europe and VIPERFAV showed broad paraspecificity. In contrast, and consistent with *V. berus* as the sole immunizing venom, ViperaTAb strongly specific for *V. berus*. While the small molecule inhibitor prinomastat nullified the effects being tested in representative venoms, DMPS failed to do so at the same molar concentrations and experimental conditions, including incubation times. In contrast to the retention of the FX activating procoagulant trait in *Vipera* species, *Montivipera* venoms were shown to be unique for the clade, exhibiting anticoagulant activity through the inhibition of FXa, paralleling its specialization for a unique montane habitat. This trend has also been noted for the lowland *V. l. gaditana*, which retained the FX activating procoagulant trait, while the derived high-altitude subspecies *V. l. latastei* had a secondary reduction in FX potency. This study therefore underscores the importance of studying evolution in parallel to venom biochemistry in order to provide data essential for understanding potential clinical effects of particular species or populations, and the relative therapeutic options.

Future work should examine for prey specific effects to reconstruct the evolutionary shaping pressures by testing several hypotheses emerging from this work regarding the selection pressures exerted by prey type, prey retaliation potential, and prey escape potential, all of which have been shown to be major drivers of venom evolution (75–77). First is the hypothesis that as mammals are particularly sensitive to procoagulant toxins due to the high circulatory rates making them rapidly subjugated by stroke as a consequence of the large blood clots formed by the venoms, the evolution larger body size is linked to an increased proportion of mammalian prey in the diet (78). This is in turn linked to a second testable hypothesis, that the greater proportion of mammals in the diet, leads to an increased chance of prey retaliation and thus a selection pressure for the increased levels of stroke-inducing procoagulant toxins in order to rapidly subjugate such dangerous prey, as has been seen for mammal specialists such as Australian elapids in the *Oxyuranus* and *Pseudonaja* genera (37). A third testable hypothesis is that the specialization into a high-altitude shift is linked to either a shift in prey type towards amphibian or reptilian prey, thus providing the selection pressure for the down-regulation of the procoagulant venom phenotype and, in the case of *Montivipera* the evolution of the anticoagulant phenotype. A linked fourth testable hypothesis is that the relative prey escape potential is another significant variable driving venom evolution, whereby montane habitats occupied by *Montivipera* and *V. l. latastei* with abundant rock cracks result in higher chance of prey escaping into inaccessible areas, versus snakes living in lowland arid habitats which are able to scent track prey over considerable distances. Such variation in relationship to altitude and prey type and prey escape potential has been noted for the rattlesnake species *Crotalus helleri* (79). In addition, as all venom samples used in this study from adult specimens, future work should examine ontogenetic shifts to ascertain if juvenile snakes have differentially procoagulant venoms, which has been noted for other species (59, 78, 80, 81).

This work thus has a broad impact, contributing to the understanding of the lethal coagulopathy produced by some species, while also providing a starting point for diverse evolutionary studies. We hope these findings stimulate further research into the evolution of venom in this group of fascinating snakes.

## Data Availability Statement

The original contributions presented in the study are included in the article/supplementary material. Further inquiries can be directed to the corresponding author.

## Ethics Statement

The studies involving human participants were reviewed and approved by UQ Human Ethics Committee Approval #2016000256 using pooled plasma from anonymous patients, supplied by the Australian Red Cross under Research Agreement #18-03QLD-0. Written informed consent for participation was not required for this study in accordance with the national legislation and the institutional requirements. The animal study was reviewed and approved by UQ NEWMA approval # 2021/AE000075.

## Author Contributions

Study conception: BF. Study design AC, CZ, ML, RC, MA, and RS. Resources ML, RC, TJ, HH, MA, and RS. Conducting of experiments. AC, CZ, and BF. Data analysis. AC, CZ, and BF. Primary draft writing AC and BF. All authors contributed to the article and approved the submitted version.

## Conflict of Interest

ML was employed by the company Ophirex, MA by Micropharm, and RS by Inosan Biopharma, all of which made products tested in this manuscript. However, the companies had no input in experimental design or reviewing of results before publication.

The remaining authors declare that the research was conducted in the absence of any commercial or financial relationships that could be construed as a potential conflict of interest.

## References

[1] Longbottom J Shearer FM Devine M Alcoba G Chappuis F Weiss DJ. Vulnerability to Snakebite Envenoming: A Global Mapping of Hotspots. Lancet (2018) 392:673–84. 10.1016/S0140-6736(18)31224-8 PMC6115328 30017551

[2] Minghui R Malecela MN Cooke E Abela-Ridder B. Snakebite-Emerging From the Shadows of Neglect. Lancet (2019) 7:e837–838. 10.1016/S2214-109X(19)30225-6/fulltext 31129124

[3] Fry BG. Snakebite: When the Human Touch Becomes a Bad Touch. Toxins (Basel) (2018) 10:1–24. 10.3390/toxins10040170 PMC5923336 29690533

[4] Fry BG Roelants K Champagne DE Scheib H Tyndall JDA King GF. The Toxicogenomic Multiverse: Convergent Recruitment of Proteins Into Animal Venoms. Annu Rev Genomics Hum Genet (2009) 10:483–511. 10.1146/annurev.genom.9.081307.164356 19640225

[5] Chippaux JP. Epidemiology of Snakebites in Europe: A Systematic Review of the Literature. Toxicon (2012) 59:86–99. 10.1016/j.toxicon.2011.10.008 22056768

[6] De Haro L Boels D. “Critical Care Toxicology,”. In: Critical Care Toxicology. Switzerland: Springer International Publishing (2020). p. 1–12. 10.1007/978-3-319-20790-2

[7] Audebert F Sorkine M Bon C. Envenoming by Viper Bites in France: Clinical Gradation and Biological Quantification by ELISA. Toxicon (1992) 30:599–609. 10.1016/0041-0101(92)90854-X 1519251

[8] Paolino G Di Nicola MR Pontara A Didona D Moliterni E Mercuri SR. *Vipera* Snakebite in Europe: A Systematic Review of a Neglected Disease. J Eur Acad Dermatol Venereol (2020) 34:2247–60. 10.1111/jdv.16722 32530549

[9] Persson H. Pathophysiology and Treatment of Envenomation by European Vipers. In: Gopalakrishnakone P Faiz S Gnanathasan C Habib A Fernando R Yang CC. (eds). Clinical Toxinology in Asia Pacific and Africa. Springer, Dordrecht (2015). 10.1007/978-94-007-6288-6_9-1

[10] Claudet I Grouteau E Cordier L Franchitto N Bréhin C. Clinical Toxicology Hyperglycemia is a Risk Factor for High-Grade Envenomations After European Viper Bites (*Vipera* Spp.) in Children. Clin Toxicol (2016) 54:34–9. 10.3109/15563650.2015.1113542 26582080

[11] Moore RS. Second-Degree Heart Block Associated With Envenomation by. Vipera berus. Arch Emerg Med (1988) 5:116–8. 10.1136/emj.5.2.116 PMC1285499 3408530

[12] Karlo R Dželalija B Župančić B Bačić I Dunatov T Kanjer A. Venomous Snakebites in the Croatian North Dalmatia Region. Cent Eur J Med (2011) 123:732–7. 10.1007/s00508-011-0085-x 22124839

[13] Marinov I Atanasov VN Stankova E Duhalov D Petrova S Hubenova A. Severe Coagulopathy After *Vipera Ammodytes Ammodytes* Snakebite in Bulgaria: A Case Report. Toxicon (2010) 56:1066–9. 10.1016/j.toxicon.2010.06.010 20600226

[14] Alencar LRV Quental TB Grazziotin FG Alfaro ML Martins M Venzon M. Diversification in Vipers: Phylogenetic Relationships, Time of Divergence and Shifts in Speciation Rates. Mol Phylogenet Evol (2016) 105:50–62. 10.1016/j.ympev.2016.07.029 27480810

[15] Siigur E Tõnismägi K Trummal K Samel M Vija H Subbi J. Factor X Activator From *Vipera Lebetina* Snake Venom, Molecular Characterization and Substrate Specificity. Biochim Biophys Acta - Gen Subj (2001) 1568:90–8. 10.1016/S0304-4165(01)00206-9 11731090

[16] Kini RM Koh CY. Metalloproteases Affecting Blood Coagulation, Fibrinolysis and Platelet Aggregation From Snake Venoms: Definition and Nomenclature of Interaction Sites. Toxins (Basel) (2016) 8:1–27. 10.3390/toxins8100284 PMC5086644 27690102

[17] Casewell NR Sunagar K Takacs Z Calvete JJ Jackson TNW Fry BG. “Snake Venom Metalloprotease Enzymes,”. In: Venomous Reptiles and Their Toxins: Evolution, Pathophysiology and Biodiscovery. New York: Oxford University Press (2015). p. 347–63.

[18] Arlinghaus FT Fry BG Sunagar KK Jackson TNW Eble JA Reeks T. “Lectin Proteins,”. In: Venomous Reptiles and Their Toxins: Evolution, Pathophysiology and Biodiscovery. New York: Oxford University Press(2015). p. 299–311.

[19] Gutiérrez JM Calvete JJ Habib AG Harrison RA Williams DJ Warrell DA. Snakebite Envenoming. Nat Rev Dis Prim (2017) 3:17063. 10.1038/nrdp.2017.63 28905944

[20] Zinenko O Stümpel N Mazanaeva L Bakiev A Shiryaev K Pavlov A. Mitochondrial Phylogeny Shows Multiple Independent Ecological Transitions and Northern Dispersion Despite of Pleistocene Glaciations in Meadow and Steppe Vipers (*Vipera Ursinii* and *Vipera Renardi*). Mol Phylogenet Evol (2015) 84:85–100. 10.1016/j.ympev.2014.12.005 25527984

[21] de Smedt J. The Vipers of Europe. JDS Verlag (2006).

[22] Pyron RA Burbrink FT Wiens JJ. A Phylogeny and Revised Classification of Squamata, Including 4161 Species of Lizards and Snakes. BMC Evol Biol (2013) 13:1–53. 10.1186/1471-2148-13-93 23627680 PMC3682911

[23] Leonardi A Fox JW Trampuš-Bakija A Križaj I. Two Coagulation Factor X Activators From *Vipera a. Ammodytes* Venom With Potential to Treat Patients With Dysfunctional Factors IXa or Viia. Toxicon (2008) 52:628–37. 10.1016/j.toxicon.2008.07.015 18760294

[24] Samel M Vija H Subbi J Siigur J. Metalloproteinase With Factor X Activating and Fibrinogenolytic Activities From *Vipera Berus Berus* Venom. Comp Biochem Physiol - B Biochem Mol Biol (2003) 135:575–82. 10.1016/S1096-4959(03)00171-4 12892749

[25] Yumiko K Toshiaki N Hisayoshi S. Isolation and Characterization of Factor X Activator From the Venom of *Vipera Aspis Aspis* . Int J Biochem (1990) 22:1053–4. 10.1016/0020-711X(90)90213-M 2282962

[26] Samel MAR Siigur J. Medium Molecular Weight Factor X Activating Enzyme From *Vipera Berus Berus* Venom. Toxicon (1995) 33:41–52. 10.1016/0041-0101(94)00143-V 7778128

[27] Garkowski A Czupryna P Zajkowska A Pancewicz Sł Moniuszko A Kondrusik M. *Vipera Berus* Bites in Eastern Poland - a Retrospective Analysis of 15 Case Studies. Ann Agric Environ Med (2012) 19:793–7. 23289356

[28] Mebert K GöçMen B İğcİ N aNil Oğuz M Kariş M UrSeNbacher S. New Records and Search for Contact Zones Among Parapatric Vipers in the Genus *Vipera* (*Barani*, *Kaznakovi*, *Darevskii*, *Eriwanensis*), *Montivipera* (*Wagneri*, *Raddei*), and *Macrovipera* (*Lebetina*). Herpetol Bull (2015) 133:13–22.

[29] De Vries A Gitter S. The Action of *Vipera Xanthina Palestinae* Venom on Blood Coagulation *In Vitro* . Brit J Haematol (1957) 3:379–86. 10.1111/j.1365-2141.1957.tb05537.x 13479687

[30] Archundia IG de Roodt AR Ramos-Cerrillo B Chippaux JP Olguín-Pérez L Alagón A. Neutralization of *Vipera* and *Macrovipera* Venoms by Two Experimental Polyvalent Antisera: A Study of Paraspecificity. Toxicon (2011) 57:1049–56. 10.1016/j.toxicon.2011.04.009 21530569

[31] Abi-Rizk A Rima M Bloquet SH Sadek R Sleiman Y Fajloun Z. Lethal, Hemorrhagic, and Necrotic Effects of *Montivipera Bornmuelleri* Venom. Curr Herpetol (2017) 36:58–62. 10.5358/hsj.36.58

[32] Ben-Ami M Hagher H Katzuni E Arens R. Recovery From Multiple Bites by *Vipera Xanthina Palestinae* . Clin Pediatr (Phila) (1982) 21:599. 10.1177/000992288202101007 7116743

[33] Cesaretli Y Ozkan O. Snakebites in Turkey: Epidemiological and Clinical Aspects Between the Years 1995 and 2004. J Venom Anim Toxins Incl Trop Dis (2010) 16:579–86. 10.1590/S1678-91992010000400007 21748231

[34] Nalbantsoy A Igci N Göçmen B Mebert K. Cytotoxic Potential of Wagner’s Viper, *Montivipera Wagneri*, Venom. North West J Zool (2016) 12:286–91.

[35] Gutiérrez JM León G Burnouf T. Antivenoms for the Treatment of Snakebite Envenomings: The Road Ahead. Biologicals (2011) 39:129–42. 10.1016/j.biologicals.2011.02.005 21429763

[36] Zdenek CN op den Brouw B Dashevsky D Gloria A Youngman NJ Watson E. Clinical Implications of Convergent Procoagulant Toxicity and Differential Antivenom Efficacy in Australian Elapid Snake Venoms. Toxicol Lett (2019) 316:171–82. 10.1016/j.toxlet.2019.08.014 31442586

[37] Zdenek CN Hay C Arbuckle K Jackson TNW Bos MHA op den Brouw B. Coagulotoxic Effects by Brown Snake (*Pseudonaja*) and Taipan (*Oxyuranus*) Venoms, and the Efficacy of a New Antivenom. Toxicol Vitr (2019) 58:97–109. 10.1016/j.tiv.2019.03.031 30910521

[38] Chowdhury A Zdenek CN Dobson JS Bourke LA Soria R Fry BG. Clinical Implications of Differential Procoagulant Toxicity of the Palearctic Viperid Genus *Macrovipera*, and the Relative Neutralization Efficacy of Antivenoms and Enzyme Inhibitors. Toxicol Lett (2021) 340:77–88. 10.1016/j.toxlet.2020.12.019 33412251

[39] Youngman NJ Chowdhury A Zdenek CN Coster K Sundman E Braun R. Utilising Venom Activity to Infer Dietary Composition of the Kenyan Horned Viper (*Bitis Worthingtoni*). Comp Biochem Physiol Part - C Toxicol Pharmacol (2021) 240:1–6. 10.1016/j.cbpc.2020.108921 33122136

[40] Stümpel N Rajabizadeh M Avci A Wüster W Joger U. Phylogeny and Diversification of Mountain Vipers (*Montivipera*, Nilson2001) Triggered by Multiple Plio-Pleistocene Refugia and High-Mountain Topography in the Near and Middle East. Mol Phylogenet Evol (2016) 101:336–51. 10.1016/j.ympev.2016.04.025 27165940

[41] Barlow A Wüster W Kelly CMR Branch WR Phelps T Tolley KA. Ancient Habitat Shifts and Organismal Diversification are Decoupled in the African Viper Genus *Bitis* (Serpentes: Viperidae). J Biogeogr (2019) 46:1234–48. 10.1111/jbi.13578

[42] Youngman NJ Debono J Dobson JS Zdenek CN Harris RJ op den Brouw B. Venomous Landmines: Clinical Implications of Extreme Coagulotoxic Diversification and Differential Neutralization by Antivenom of Venoms Within the Viperid Snake Genus. Bitis. Toxins (Basel) (2019) 11:1–20. 10.3390/toxins11070422 PMC6669450 31331004

[43] Halassy B Brgles M Habjanec L Balija ML Kurtović T Marchetti-Deschmann M. Križaj IIntraspecies Variability in *Vipera Ammodytes Ammodytes* Venom Related to Its Toxicity and Immunogenic Potential. Comp Biochem Physiol - C Toxicol Pharmacol (2011) 153:223–30. 10.1016/j.cbpc.2010.10.007 20971215

[44] Malina T Krecsák L Westerström A Szemán-Nagy G Gyémánt G M-Hamvas M. Individual Variability of Venom From the European Adder (*Vipera Berus Berus*) From One Locality in Eastern Hungary. Toxicon (2017) 135:59–70. 10.1016/j.toxicon.2017.06.004 28602828

[45] Ferquel E de Haro L Jan V Guillemin I Jourdain S Teynié A. Reappraisal of *Vipera Aspis* Venom Neurotoxicity. PloS One (2007) 2:1–18. 10.1371/journal.pone.0001194 PMC2065900 18030329

[46] Petras D Hempel BF Göçmen B Karis M Whiteley G Wagstaff SC. Intact Protein Mass Spectrometry Reveals Intraspecies Variations in Venom Composition of a Local Population of *Vipera Kaznakovi* in Northeastern Turkey. J Proteomics (2019) 199:31–50. 10.1016/j.jprot.2019.02.004 30763806 PMC7613002

[47] Arikan H Göçmen B İğci N Akman B. Age-Dependent Variations in the Venom Proteins of Vipera Kaznakovi Nikolsky, 1909 and *Vipera Ammodytes* (Linnaeus, 1758) (Ophidia: Viperidae). Turkish J Zool (2014) 38:216–21. 10.3906/zoo-1303-17

[48] Oulion B Dobson JS Zdenek CN Arbuckle K Lister C Coimbra FCP. Factor X Activating *Atractaspis* Snake Venoms and the Relative Coagulotoxicity Neutralising Efficacy of African Antivenoms. Toxicol Lett (2018) 288:119–28. 10.1016/j.toxlet.2018.02.020 29462691

[49] Rogalski A Soerensen C op den Brouw B Lister C Dashvesky D Arbuckle K. Differential Procoagulant Effects of Saw-Scaled Viper (Serpentes: Viperidae: *Echis*) Snake Venoms on Human Plasma and the Narrow Taxonomic Ranges of Antivenom Efficacies. Toxicol Lett (2017) 280:159–70. 10.1016/j.toxlet.2017.08.020 28847519

[50] Casewell NR Wagstaff SC Wus̈ter W Cook DAN Bolton FMS King SI. Medically Important Differences in Snake Venom Composition are Dictated by Distinct Postgenomic Mechanisms. Proc Natl Acad Sci USA (2014) 111:9205–10. 10.1073/pnas.1405484111 PMC4078820 24927555

[51] Debono J Bos MHA Frank N Fry B. Clinical Implications of Differential Antivenom Efficacy in Neutralising Coagulotoxicity Produced by Venoms From Species Within the Arboreal Viperid Snake Genus. Trimeresurus. Toxicol Lett (2019) 316:35–48. 10.1016/j.toxlet.2019.09.003 31509773

[52] Isbister GK Duffull SB Brown SGA. Failure of Antivenom to Improve Recovery in Australian Snakebite Coagulopathy. QJM (2009) 102:563–8. 10.1093/qjmed/hcp081 19570990

[53] Kurtović T Brvar M Grenc D Lang Balija M Križaj I Halassy B. A Single Dose of ViperfavTM may be Inadequate for *Vipera Ammodytes* Snake Bite: A Case Report and Pharmacokinetic Evaluation. Toxins (Basel) (2016) 8:244. 10.3390/toxins8080244 PMC4999860 27548220

[54] Bittenbinder MA Zdenek CN Op Den Brouw B Youngman NJ Dobson JS Naude A. Coagulotoxic Cobras: Clinical Implications of Strong Anticoagulant Actions of African Spitting *Naja* Venoms That Are Not Neutralised by Antivenom But are by LY315920 (Varespladib). Toxins (Basel) (2018) 10:1–12. 10.3390/toxins10120516 PMC6316626 30518149

[55] Lister C Arbuckle K Jackson TNW Debono J Zdenek CN Dashevsky D. Catch a Tiger Snake by Its Tail: Differential Toxicity, Co-Factor Dependence and Antivenom Efficacy in a Procoagulant Clade of Australian Venomous Snakes. Comp Biochem Physiol Part - C Toxicol Pharmacol (2017) 202:39–54. 10.1016/j.cbpc.2017.07.005 28757215

[56] Sousa LF Nicolau CA Peixoto PS Bernardoni JL Oliveira SS Portes-Junior JA. Comparison of Phylogeny, Venom Composition and Neutralization by Antivenom in Diverse Species of *Bothrops* Complex. PloS Negl Trop Dis (2013) 7:1–16. 10.1371/journal.pntd.0002442 PMC3772048 24069493

[57] Sousa LF Zdenek CN Dobson JS op den Brouw B Coimbra FCP Gillett A. Coagulotoxicity of *Bothrops* (Lancehead Pit-Vipers) Venoms From Brazil: Differential Biochemistry and Antivenom Efficacy Resulting From Prey-Driven Venom Variation. Toxins (Basel) (2018) 10:1–23. 10.3390/toxins10100411 PMC6215258 30314373

[58] Bourke LA Zdenek CN Neri-Castro E Bénard-Valle M Alagón A Gutiérrez JM. Pan-American Lancehead Pit-Vipers: Coagulotoxic Venom Effects and Antivenom Neutralisation of *Bothrops Asper* and *B. Atrox* Geographical Variants. Toxins (Basel) (2021) 13:1–18. 10.3390/toxins13020078 PMC7911261 33499001

[59] Seneci L Zdenek CN Chowdhury A Rodrigues CFB Neri-Castro E Bénard-Valle M. A Clot Twist: Extreme Variation in Coagulotoxicity Mechanisms in Mexican Neotropical Rattlesnake Venoms. Front Immunol (2021) 12:612846. 10.3389/fimmu.2021.612846 33815366 PMC8011430

[60] Xie C Albulescu L-O Bittenbinder MA Somsen GW Vonk FJ Casewell NR. Neutralizing Effects of Small Molecule Inhibitors and Metal Chelators on Coagulopathic *Viperinae* Snake Venom Toxins. Biomedicines (2020) 8:1–18. 10.3390/biomedicines8090297 PMC7555180 32825484

[61] Di Nicola MR Pontara A Kass GEN Kramer NI Avella I Pampena R. Vipers of Major Clinical Relevance in Europe: Taxonomy, Venom Composition, Toxicology and Clinical Management of Human Bites. Toxicology (2021) 453:152724. 10.1016/j.tox.2021.152724 33610611

[62] Leonardi A Sajevic T Pungerčar J Križaj I. Comprehensive Study of the Proteome and Transcriptome of the Venom of the Most Venomous European Viper: Discovery of a New Subclass of Ancestral Snake Venom Metalloproteinase Precursor-Derived Proteins. J Proteome Res (2019) 18:2287–309. 10.1021/acs.jproteome.9b00120 PMC6727599 31017792

[63] Bocian A Urbanik M Hus K Łyskowski A Petrilla V Andrejčáková Z. Proteome and Peptidome of *Vipera Berus Berus* Venom. Molecules (2016) 21:1–13. 10.3390/molecules21101398 PMC6274168 27775574

[64] Giribaldi J Kazandjian T Amorim FG Whiteley G Wagstaff SC Cazals G. Venomics of the Asp Viper *Vipera Aspis Aspis* From France. J Proteomics (2020) 218:1–10. 10.1016/j.jprot.2020.103707 32087377

[65] Hempel B-F Damm M Mrinalini ∇ Gö Ç Men B Karış M. Extended Snake Venomics by Top-Down in-Source Decay: Investigating the Newly Discovered Anatolian Meadow Viper Subspecies, *Vipera Anatolica Senliki* . J Proteome Res (2020) 19:1731–49. 10.1021/acs.jproteome.9b00869 32073270

[66] Göçmen B Heiss P Petras D Nalbantsoy A Süssmuth RD. Mass Spectrometry Guided Venom Profiling and Bioactivity Screening of the Anatolian Meadow Viper, *Vipera Anatolica* . Toxicon (2015) 107:163–74. 10.1016/j.toxicon.2015.09.013 26385313

[67] Kovalchuk S Ziganshin R Starkov V Tsetlin V Utkin Y. Quantitative Proteomic Analysis of Venoms From Russian Vipers of *Pelias* Group: Phospholipases A2 are the Main Venom Components. Toxins (Basel) (2016) 8:105. 10.3390/toxins8040105 27077884 PMC4848631

[68] Alekseeva AS Tretiakova DS Chernikov VP Utkin YN Molotkovsky JG Vodovozova EL. Heterodimeric *V. Nikolskii* Phospholipases A2 Induce Aggregation of the Lipid Bilayer. Toxicon (2017) 133:169–79. 10.1016/j.toxicon.2017.05.015 28528175

[69] Zinenko O Tovstukha I Korniyenko Y. PLA2 Inhibitor Varespladib as an Alternative to the Antivenom Treatment for Bites From Nikolsky’s Viper *Vipera Berus Nikolskii* . Toxins (Basel) (2020) 12:1–7. 10.3390/toxins12060356 PMC7354479 32485836

[70] Latinović Z Leonardi A Koh CY Kini RM Trampuš Bakija A Pungerčar J. The Procoagulant Snake Venom Serine Protease Potentially Having a Dual, Blood Coagulation Factor V and X-activating Activity. Toxins (Basel) (2020) 12:1–16. 10.3390/toxins12060358 PMC7354534 32485989

[71] Leonardi A Sajevic T Latinović Z Pungerčar J Lang Balija M Trampuš Bakija A. Structural and Biochemical Characterisation of VaF1, a P-IIIa Fibrinogenolytic Metalloproteinase From *Vipera Ammodytes Ammodytes* Venom. Biochimie (2015) 109:78–87. 10.1016/j.biochi.2014.12.013 25549999

[72] Karabuva S Lukšić B Brizić I Latinović Z Leonardi A Križaj I. Ammodytin L is the Main Cardiotoxic Component of the *Vipera Ammodytes Ammodytes* Venom. Toxicon (2017) 139:94–100. 10.1016/j.toxicon.2017.10.003 29030107

[73] Karabuva S Brizić I Latinović Z Leonardi A Križaj I Lukšić B. Cardiotoxic Effects of the *Vipera Ammodytes Ammodytes* Venom Fractions in the Isolated Perfused Rat Heart. Toxicon (2016) 121:98–104. 10.1016/j.toxicon.2016.09.001 27623431

[74] Georgieva D Risch M Kardas A Buck F Von Bergen M Betzel C. Comparative Analysis of the Venom Proteomes of *Vipera Ammodytes Ammodytes* and *Vipera Ammodytes Meridionalis* . J Proteome Res (2008) 7:866–86. 10.1021/pr070376c 18257516

[75] Yang DC Deuis JR Dashevsky D Dobson J Jackson TNW Brust A. The Snake With the Scorpion’s Sting: Novel Three-Finger Toxin Sodium Channel Activators From the Venom of the Long-Glanded Blue Coral Snake (Calliophis Bivirgatus). Toxins (Basel) (2016) 8:1–21. 10.3390/toxins8100303 PMC5086663 27763551

[76] Harris RJ Zdenek CN Harrich D Frank N Fry BG. An Appetite for Destruction: Detecting Prey-Selective Binding of α-Neurotoxins in the Venom of Afro-Asian Elapids. Toxins (Basel) (2020) 12:1–12. 10.3390/toxins12030205 PMC7150784 32210072

[77] Holding ML Strickland JL Rautsaw RM Hofmann EP Mason AJ Hogan MP. Phylogenetically Diverse Diets Favor More Complex Venoms in North American Pitvipers. Proc Natl Acad Sci USA (2021) 118:1–10. 10.1073/pnas.2015579118/-/DCSupplemental.y PMC8092465 33875585

[78] Jackson T Koludarov I Ali S Dobson J Zdenek C Dashevsky D. Rapid Radiations and the Race to Redundancy: An Investigation of the Evolution of Australian Elapid Snake Venoms. Toxins (Basel) (2016) 8:1–24. 10.3390/toxins8110309 PMC5127106 27792190

[79] Sunagar K Undheim EAB Scheib H Gren ECK Cochran C Person CE. Intraspecific Venom Variation in the Medically Significant Southern Pacific Rattlesnake (*Crotalus Oreganus Helleri*): Biodiscovery, Clinical and Evolutionary Implications. J Proteomics (2014) 99:68–83. 10.1016/j.jprot.2014.01.013 24463169

[80] Furtado MFD Maruyama M Kamiguti AS Antonio LC. Comparative Study of Nine *Bothrops* Snake Venoms From Adult Female Snakes and Their Offspring. Toxicon (1991) 29:219–26. 10.1016/0041-0101(91)90106-2 1646500

[81] Antunes TC Yamashita KM Barbaro KC Saiki M Santoro ML. Comparative Analysis of Newborn and Adult *Bothrops Jararaca* Snake Venoms. Toxicon (2010) 56:1443–58. 10.1016/j.toxicon.2010.08.011 20816886

